# The Conversazione at Burlington House

**Published:** 1920-05-29

**Authors:** 


### THE CONVERSAZIONE AT BURLINGTON HOUSE.

The mere doctor who goes to a conversazione given by the Royal Society has his breath taken away as he realises his hopeless ignorance and the small extent to which he can keep up with the trend of general scientific knowledge. Such was our first impression on going to Burlington House on the 12th of this month, but after we had again begun to breathe we became intensely interested in all that we saw, and almost surprised at the extent to which the various sciences touched upon the fringes of our work, even when they were not directly connected with it.

### The Rise of Life.

Dr. W. H. Spencer had one of those interesting exhibits which link up the present day with the rise of life in the early Palæozoic times. He showed how the starfish of these early days were similar to those of to-day, how some were carnivorous and some herbivorous, and here he took up the work of the Danish Fishery Board described by Dr. Petersen. The Board has studied the habits of starfish in the detritus at the bottom of the sea beyond the belt in which the sea grasses grow. It has made certain estimates in the amount of herbivorous food which must be taken to form unit mass of a new organism, and has compared with this the amount of animal food which must be taken to produce a similar mass. From these experiments a glimmering of practical application in the production of fish food is shining.

From the Palæozoic age we jumped to the Miocene and saw the plaster casts of an atlas and another cervical vertebra of a rhinoceros which had been brought from Baluchistan by Mr. C. Forster Cooper, who had also brought along the corresponding bones of the present-day rhinoceros. It is almost impossible to imagine how enormous this beast must have been. We made no measurement on the spot, but believe that we are not exaggerating when we say that the bones of the pigmy descendant of to-day were not more than one quarter in each dimension of those of his ancestor. Think of our lazy and ugly friend of the Zoo, and then try to reconstruct sixty-four such masses into a rolling mass of animal tissues. The weight of bone to be carried by such an animal is enormous, and in the specimen shown some compensation was made for this by the expansion of the vertebral canal into the subsidence of the osseous tissue, so that the bones are hollowed out from either side.

### Ancient Man.

Another jump in time brought us down to Palæolithic man, of whose implements Mr. L. Treacher showed an enormous sample from near Maidenhead. How this was used is hard to say; no modern man could wield it with one hand, and would find difficulty in using it if fitted to a shaft as a spear. An onlooker allowed his imagination to rove, and suggested that even in these days man had a Parliament, and that this was the mace. Neolithic man was represented by specimens shown by Mr. S. H. Warren of axes from a factory at Penmaenmawr. The exhibitor says "Axes are found in every stage of manufacture, discarded on account of breakage or unsatisfactory shape." We must admit that this was a new idea to us. We had always imagined each Neolithic man chipping his own flint and making his own arms, and had never thought that social life had advanced so far as the differentiation of labour or of place to one occupation.

Mr. A. V. Hill, F.R.S., showed an instrument for recording the production of heat in muscles, and Dr. J. H. Mummery showed a microscopical specimen in which he claimed that he had demonstrated some nerve end-cells in the pulp of the human tooth. In so slight an examination as we were able to make, we are not prepared to admit that the structures demonstrated were nerve-cells; but if Dr. Mummery's find proves to be such, he has discovered a structure entirely new to human and to animal anatomy; such nerve cells are unknown in connection with the sensory system in any other part of the body.

### Preventive Medicine.

Three interesting exhibits bear some relation to preventive medicine. Samples of mite-infested flora were shown, and gave suggestions as to how such infestation might be prevented. Professor Nuttall and Dr. Keilin showed specimens of Hermaphroditism in Pediculus Humanus. There are few of us who are uninterested in this subject. We do not think it is an exaggeration to say that the pediculus caused more loss of man-power to our armies than any other factor, not excluding enemy shells. If it were possible to estimate the loss of man-power in man-day units, not merely of stay in hospital but of being off duty or on light duty, we should find those of diseases of which the pediculus is either the cause or a determining factor far outstripped any other group. A full study of the life-history of the pediculus may help us to diminish these causes of sickness in a future war.

### The Physical Sciences.

So far we have considered only the exhibits in connection with the biological sciences. All were interested in the demonstration of wireless telephony by Mr. Campbell Swinton, who caused us to hear a gramophone being played at Chelmsford as modified by Morse code signals picked up from Poldhu. Mr. Edwin Fraser afforded us some hope for the future by suggesting that coal might be recovered from waste dumps, and Messrs. Crossfield and Sons, with their synthetic products for perfumery, made us wonder whether our grandchildren were to rave over the relative sweetness of various esthers of capriotic acid rather than over "essences distilled from the fragrance of the East." The Air Ministry Laboratory, the National Physical Laboratory, the Meteorological Office, the Admiralty Compass Department, and the Royal Geographical Society all showed exhibits which demonstrate the increasing application of many sciences to flight, and Major G. W. C. Kaye showed how the $x$-rays may help in preventive as well as in clinical surgery.

### The Application of Science.

If we had space we would willingly note other things that we saw during this most interesting evening. One thing we must add. What impressed us most was the fact that in every branch science was being applied to every-day life and was being brought to the help of the practical man in all that he does. The dreamy professor whose chief delight in his discovery is that it cannot possibly be of any use to anybody may still be in existence. He was not in evidence at the Royal Society on May 12. Nor do we believe that the man of science has in any way given up his ideal of knowledge for the sake of knowledge.

